# Diabetes

**Published:** 1904-12-24

**Authors:** 


### Progress in Medicine and Surgery.

### DIABETES.

(*Concluded from page* 209.)

**Treatment.**—For purposes of treatment recent writers divide the cases into three classes: (1) mild, in which the sugar disappears on rigid carbohydrate-free diet; (2) medium, in which the sugar does not disappear, but no acetone bodies are found in the urine; (3) severe, in which, in addition to sugar, acetone bodies occur in the urine, and the excretion is not controlled by rigid diet. Williamson[11] remarks that to treat a given case satisfactorily the form of diabetes must be determined by preliminary dieting and examination of the urine, and the existence or not of phthisis, heart disease, and neuritis ascertained. The weight and sugar excretion must be accurately watched and the bowels regulated.

*Diet.*—Williamson advises in mild cases a rigid diet for a certain period, after which small amounts of carbohydrate are added till the amount which the patient is able to take without sugar excretion is found. In more severe cases, especially if the weight decreases after some months of rigid diet, some carbohydrate in the form of bread must be allowed, but the sugar excretion should be kept at about 500 grains per diem. In the most severe form a very rigid diet is also sometimes injurious, and in these cases, though sugar is to be avoided, some starchy foods may be allowed. Hutchison,[12] while advocating dieting on very similar lines, points out that the reason for treating what is apparently only a symptom is twofold: by diminishing the amount of sugar in the blood the occurrence of irritative complications, such as neuritis, cataract, polyuria, and thirst, and lesions due to micro-organisms such as carbuncle are obviated, and the tissues which are unequal to dealing with carbohydrates are rested and given a chance of recuperating and regaining some of their lost function. In the mild cases he advises carbohydrates to an amount well under that at which sugar appears in the urine, in order not to overtax carbohydrate metabolism; in medium cases a strict diet, which if convenient may be suddenly adopted and should be continued as long as possible; in severe cases a very gradual adoption of carbohydrate-free food, so as not to risk the supervention of coma. Moreover, in these cases sugar may be formed from proteid; more than 500 grains of proteid should not therefore be given per diem, and the diet made up with small amounts of carbohydrate food. Shattuck [13] insists on the necessity of individualising the treatment, and not dieting all patients rigidly alike. Mild cases may be kept on rigid diet two or three months, and two or three ounces of bread then allowed to test the effect. More severe cases must be allowed bread only with extreme caution; in the most severe a fixed amount of bread is always to be allowed. He advises also a day of starvation in some cases, every two or three months. As regards special foods Williamson [14] advocates starch as being less injurious than sugar, with which Hutchison agrees. He points out that many of the older bread substitutes are useless, as they are unpalatable, expensive, and contain a good deal of starch. He advocates protene bread, which is best made at home. Four ounces of protene No. 2 are beaten thoroughly with two ounces of butter and two eggs; eight small "cobs" are made of this and baked. Casoid and casoid meal are also reliable preparations. Aleuronat biscuits are also valuable, and cocoanut cakes may be allowed as the amount of sugar they contain is very small and nearly entirely decomposed by the yeast used to make the cakes "rise." Cream is valuable, and ordinary milk may be allowed freely if it has no effect on the sugar excretion. When ordinary milk cannot be taken a substitute can be made as follows: four tablespoonfuls of cream are well mixed with a pint of water and allowed to stand in a cool place. The sugar remains in the water when the fat has risen, and the latter is skimmed off and added to water in which the white of an egg, a little salt, and a trace of saccharin have been mixed. As to beverages beer is most injurious, and spirits least so, but these should be taken in great moderation, especially in the milder forms. Shattuck [15] advocates a dry champagne, whisky, or brandy, Moselle, or a Californian light hock. He does not advocate the use of diabetic breads. Hutchison [16] points out the great value of fat to the diabetic, in the form of butter, bacon, Devonshire cream, or salad oil. At least $\frac{1}{4}$ lb. of butter should be taken daily. He thinks a little alcohol often aids the digestion of fat. In strict diets, oysters and liver must be avoided, and sausages are always too uncertain in composition to be allowable. Milk may be given in some cases, in others a diabetic milk must be used. He advocates casoid-meal bread and warns practitioners against ever allowing toast, which, in spite of a widespread idea to the contrary, contains weight for weight more starch than ordinary bread. Potatoes may be allowed in many cases, as they are not very starchy and are an excellent vehicle for butter. Nuts are useful, as containing much fat; fresh fruits must be cautiously used, and dried or preserved fruits not at all. In elderly people with mild attacks it is often enough to forbid sugar and limit starch; in these, alcohol must be given with caution. Sawyer,[17] following Mossé, advocates potatoes for diabetics, as having a distinctly beneficial effect owing to the amount of potash and phosphoric acid they contain. They must, however, be always steamed in their skins to avoid losing the salts. He also prepares a kind of flour from potatoes, which can be made into biscuits or, mixed with bran, yeast, eggs, and butter, into an excellent bread substitute. Barr,[18] while agreeing as to the value of potatoes, thinks they should be baked in their jackets and served with plenty of butter. He cautions against the use of too much potato, owing to the starch it contains, and thinks the maximum should be one potato a day. Thorne [19] does not advise the addition of bran, as it is capable of exercising mechanical irritation and setting up gastro-duodenal catarrh. He also thinks that soda-water should be excluded, though allowed by Sawyer and Hutchison, as being a rubefacient to the gastric mucous membrane and liable to cause undue distension of the stomach by gas. Van Noorden [20] believes that oatmeal has a distinct effect in decreasing sugar excretion. It is boiled for a long time, and then butter, egg- or vegetable-albumen, and a little salt are added. A soup containing 250 gm. oats, 100 gm. albumen, 300 gm. butter is the total allowance per diem. It is given every two hours, and a little brandy or wine and some strong black coffee allowed in addition. Only a few cases, however, can be treated in this way, and they appear to be the severe ones, and not mild cases without acetonuria.

*Drugs.*—Williamson [21] advocates salicylate of soda and aspirin as having a marked effect in many cases in diminishing sugar excretion. They are not specifics, and are useless in severe cases; in many mild cases, however, they enable more carbohydrate to be added to the diet without the appearance of sugar in the urine. Marked intolerance of the drug is, of course, a contraindication. Aspirin is, however, better borne than salicylates. He usually begins with 10 grains of the latter thrice daily, increasing up to 15 grains, three, four, or five times a day if necessary. Aspirin may be given in 10-grain doses increased to 15, at first twice and then up to four or five times daily. The powder should be taken in water with a little lemon-juice, and alkalies or soda-water avoided as they decompose the drug. Galloway [22] has obtained good results with potassium iodide, which he employs in all cases; 10 or 15 grains are given every three or four hours till the sugar disappears, and the drug is then stopped for a few days or until sugar is again found. It is then resumed in smaller doses, and the process repeated until sugar finally disappears. Alcohol in all forms and sugar are to be avoided, but otherwise the diet should be rich, plentiful and varied. Thompson [23] only regards drugs as useful in meeting special symptoms. He has found cod-liver oil, iron and certain intestinal antiseptics of value. Crofton,[24] finding that pancreatic extract did not destroy sugar outside the body, tried a mixed extract of pancreas, muscle and blood, which he found had a marked effect on saccharin solutions. In diabetic patients he also found this extract in large doses (over one ounce) diminished the output of sugar, and allowed a larger amount of bread to be assimilated. He quotes six cases. The patients' weights were well kept up.

General Treatment.—Williamson [25] finds that mild cases are often benefited by a course of waters at Carlsbad, which he thinks is due to the drinking of those waters hot from the spring. In bad cases, however, such a course only does harm.

Shattuck[26] also advises change of climate during winter. He also points out the importance of protecting the patient against cold, and the bad effects of worry and anxiety. Moderate exercise should be taken and the skin and excretory organs attended to.

**Coma.**—Hutchison advocates a diet of skimmed milk given freely, either plain or mixed with Vichy water. Alcohol should also be given freely, and bicarbonate of soda added to the milk. Douglas[27] recommends an alkali, either large doses by the mouth, or, better, by subcutaneous or intravenous injection. He also mentions Schwartz's case, in which on two occasions 50–70 grammes of gluconic acid in alkaline water, together with 140-gramme doses of bicarbonate of soda, were successful; while on a third occasion, in the same patient, the latter alone was ineffectual. Lépine's treatment by phenol has not been successful, though antipyrin and phenacetin have been used a good deal in France. Rolleston and Tebb[28] note that in a case of Owen's, acetone bodies disappeared completely within an hour after the transfusion of four pints of salt solution containing $1\frac{1}{2}$ drachm of bicarbonate to the pint, but in their own cases small doses of the drug were ineffectual, and morphia appeared equally useless. In one of Dreschfeld and Moore's cases large doses of alkalies and aperients relieved symptoms of coma due to acid intoxication.

[11] Lancet, Jan. 23, 1904, p. 213. [12] Practitioner, June, 1904, p. 800. [13] Med. Rec., May 21, 1904, p. 817. [14] *Loc. cit.* [15] *Loc. cit.* [16] *Loc. cit.* [17] Brit. Med. Jour., March 5, 1904, p. 537. [18] Ibid., March 19, 1904, p. 693. [19] Ibid. [20] Med. Rec., May 28, 1904, p. 885. [21] Lancet, Jan. 23, 1904, p. 214. [22] Med. Rec., April 16, 1904, p. 628. [23] Ibid., May 21, 1904, p. 817. [24] Edin. Med. Clin. Jour., July, 1904, p. 85. [25] *Loc. cit.* [26] *Loc. cit.* [27] *Loc. cit.* [28] *Loc. cit.*, p. 115.

